# Six Years’ Work in Oral Prophylaxis

**Published:** 1905-03

**Authors:** 


### Reviews of Dental Literature.

SIX YEARS' WORK IN ORAL PROPHYLAXIS.[1] By D. D. Smith, D.D.S., M.D., Philadelphia, Pa.

The term oral prophylaxis, so recently injected into the vocabulary of dentistry, is endowed with a meaning which as yet seems not well understood.

Prophylaxis is quite commonly confounded with prophylactic,

---

[1] Read before the New Jersey State Dental Society, Thirty-fourth Annual Session, July 20, 1904.

and is used by speakers and writers interchangeably with it, when, in fact, the two words have quite separate and distinctive significations. These terms are also used, apparently without discrimination, as synonyms for asepsis, oral hygiene, oral massage, and whatever is supposed to ward off disease of the teeth.

One dental writer says, "Prophylaxis has invaded the domain of nearly every disease, and its science and practice is daily increasing and its field of usefulness becoming better known."

Prophylactic measures may be employed to ward off some diseases when preventive remedies, from experience, commend themselves, or when experimentation in this direction seems warranted, but to affirm that "*prophylaxis* has invaded the domain of nearly every disease" seems a confusion of terms.

It is to be hoped that the science of prophylaxis is becoming better known, but it cannot at present be said that prophylactic remedies or the prophylaxis treatment is "well understood" in dentistry, or that either is applied or practised as a science.

In defining the terms prophylactic and prophylaxis we would emphasize the fact that prophylactic is a word used both as an adjective and as a noun, and that it relates to therapeutic *remedies*, which may be administered as preventive of disease. Prophylaxis relates to remedial or preventive *treatment*. The dictionaries generally pointed to the signification as here given when the first paper on this subject was prepared and read in 1898, under the caption "Prophylaxis in Dentistry." Only one has been found to give prophylaxis any other meaning; this is as follows: "Prophylaxis, preservative treatment for disease; conservation;" from which one would naturally infer that prophylaxis preserves and conserves disease, instead of preventing it.

Webster's Dictionary, 1901 edition, defines prophylactic as follows: "Prophylactic, a *medicine* which preserves or defends against disease; a preventive;" and "prophylaxis, the *art* of preserving from, or of preventing, disease. The observance of the rules necessary for the preservation of health; preservative or *preventive treatment.*"

Worcester gives: "Prophylactic, an adjective, preventing disease; preventive; preservative." Prophylaxis not defined.

The Century: "Prophylaxis, *n.*, anything, as a *medicine*, which defends against disease; a preventive of disease;" and "prophylactic, *a.*, preventive; defending from disease, as prophylactic doses of quinine." (That is, treatment by therapy; therapeutic treatment.) "Prophylaxis, the guarding against the attack of some disease;" example: "The germs do not appear to be very tenacious of life, so that an efficient prophylaxis can be readily exercised.—*Science.*"

Standard Dictionary: "Prophylactic, any medicine or measure efficacious in protecting from disease." "Prophylaxis, preservative or preventive *treatment* for disease; especially a particular form of disease in an individual."

Briefly then, prophylaxis is an art or a surgical treatment, involving manipulative effort, as distinguished from the administration of a systemic medicament or a therapeutic remedy. Hence, oral prophylaxis implies surgical instrumentation or treatment of the mouth and teeth in contradistinction to a germicide, a wash, or any form of medication for the prevention of disease in the oral cavity.

Although the title of this paper is "Six Years' Work in Oral Prophylaxis," the time really covered in developing the treatment, by the author, embraces a period of ten years. Experiments were carried forward on members of my own family and among friends for four years prior to the reading of the paper on "Prophylaxis in Dentistry" referred to above, that theories might be verified before giving any publicity to the treatment. The results were so satisfactory that in the beginning of the fifth year it was determined to force recognition of it in general practice, little dreaming what astounding revelations were to follow.

In reviewing the subject at this time it will be my endeavor, as in former papers, to present some of the remarkable phenomena which appear as unvarying results, from a consistent and intelligent prophylaxis treatment of the mouth and teeth. While these results are surprising, almost startling, to one who has never witnessed them, they are but the logical and inevitable outcome of this system of caring for the mouth and teeth.

### PROPHYLAXIS TREATMENT AS APPLIED TO TEETH IN THE MOUTH.

To understand what the prophylaxis treatment in its application to the mouth and teeth is, we must understand the long train of unstudied and disregarded pathologic conditions which have their origin in the undisturbed infection on and about the teeth in the human mouth.

Calcific deposits are constantly occurring and recurring, and not less the more immediately hurtful acidulated bacterial accumulations; and more dangerous still the inspissated mucus which cements mouth fluids, mucoid excretions, decomposing food particles, and other septic and odoriferous matter upon the teeth; this débris is all maintained in the high normal heat of the mouth—98.6° F.—and furnishes ideal conditions for the proliferation of germs, the induction of decay, and the fostering of disease in the human system. The pathologic states of the mouth and teeth, in the order of their seriousness and frequency, may be defined as follows: pericemento-alveolar inflammations, dental caries, and impeded alveolar development.

Resulting from the first and most important—pericemento-alveolar inflammations—we find gum inflammations, alveolar absorption, pyorrhœa, stomatitis, and pericemental abscess; the latter a serious condition, resulting in the inevitable loss of the tooth. Caries, with its attendant decalcification of dentine, followed by pulp exposure, alveolar abscess, and final destruction of the *crown* of the tooth, although it has engrossed the thought of dentistry in the past and is the embodiment of its efforts in the present, is far less serious in its results. Impeded alveolar development, a common result of mouth infection of childhood, is a cause of many alveolar deformities and many crowded and irregular conditions of the teeth;—conditions which detract from the charm of facial expression, greatly increase infection, incite decay and multiply loss of the teeth.

Whether conscious of it or not, every human being with natural teeth is a sufferer from mouth infection; some to a greater extent than others—the civilized more than the savage.

The oral prophylaxis treatment, as instituted and recommended by the author, has for its object and aim the freeing of the oral cavity of conditions of tooth decay which has become practically universal, and the eradication of infection from the same, to conserve human health and prolong human life. In all stations and conditions the treatment has demonstrated its sufficiency and its reasonable practicability. The benefits accruing to the mouth and teeth, and thence to the general health, are startling in the scope of their marvellous efficiency.

SUBSTANTIATION OF THEORIES RESPECTING TOOTH DECAY AND TOOTH PRESERVATION.

In previous writings I have persistently maintained that tooth decay always begins on some surface of the tooth which is exposed to the fluids of the mouth. It never originates in the substance of the tooth unexposed, either crown or root. If the decay is in the crown, it begins in the enamel; if devoid of enamel, it may attack the surface of the dentine at any point. Once in the dentine, the decay proceeds along the lines of the tubules in the direction of the pulp. The process of tooth-solution, which we call decay, is largely chemical, and is hindered or opposed by two conditions only: (*a*) the composition or consolidation of enamel and dentine; (*b*) the expression of vital energy interposed by a living pulp—a force often scarcely appreciable. No matter what the physical state of the crown of the tooth, whether hard or soft, good or bad, alive or dead, the agents and agencies which cause decay are in the environment or surroundings of the teeth. Evidence from practical experience in support of this is abundant and unvarying. A simple and incontestable proof is found in the state of an extracted tooth. Let a pulpless, decaying tooth be removed from a mouth where the environments are such that resolution is rapidly taking place, and let it be placed in water, alcohol, or glycerin, or simply exposed to the air, and all decay in that tooth is immediately arrested; further disintegration will come only with the lapse of years.

To institute simulated or artificial decay in a tooth while it is out of its natural environment, as has been frequently done, is wholly unavailing as a matter of scientific investigation. All such processes are but artificial decomposition, and are so unlike tooth-decay in the mouth that true scientific research is not advanced by them. Teeth are the subject of true decay only when they are in the normal menstruum and temperature of the mouth.

The all-important matter for dentistry to consider and determine is, whether oral prophylaxis, understood and properly instituted, will arrest, prevent, or retard decay of the teeth *in the mouth.* To this end there is needed not *theorizing* but clear clinical observation and close study of mouth conditions.

Ten years' experimentation has abundantly demonstrated that while the treatment will not arrest decay which is already in progress, whether as new cavities or under old or imperfect fillings, it will prevent its appearance on surfaces where the prophylaxis treatment has been regularly instituted. It will also greatly retard the progress of decay in open cavities and under old fillings.

An editorial in a recent number of *American Medicine* reads in part as follows: "There are ten millions of them! Everybody knows about them, the disease they spread, their horrors, their worse than loathsomeness! Every one endures, submits in silence, feels himself powerless to remedy. Boards of health cannot, or think they cannot, attack the evil; or they are too busy with things they think are more important. And so the filthy country and village water-closet persists from generation to generation. The intellectual philanthropist is yet to come who shall undertake one of the greatest reforms of the world!"

The greatest sanitary reform of the world is not the abolition of the village closet, but it lies in the herculean task of revolutionizing the unsanitary and infectious condition of the human mouth. Contagion and disease from the latter are a thousand-fold more subtle and dangerous than from the former, for infection in the human mouth is found not in country and village alone, but in town and city—in all places where humanity dwells.

If this be true,—and who shall dispute it?—oral prophylaxis treatment presents itself as a subject of momentous import both for the profession and the public. It is not a matter to be tossed hastily aside because it does not coincide with our prejudices or preconceived opinions; neither is it a matter to be adjudged wholly from the stand-point of self-interest. Theoretical difficulties have been presented and unjustifiable self-interests have been urged against it, until we have sometimes asked with unutterable groanings, "Is dentistry, with such views, worthy to be called a profession?"

One writer declares, "The great limitation to the universal success of Dr. Smith's method lies in the non-receptiveness of the mass of people to methods involving as much attention to detail as must be required of those patients following his system."

This unjustifiable deduction in the *Dental Cosmos*, from the pen of a professor in dentistry, exhibits not only the prejudice of inexperience, but shows a lamentable, if not inexcusable, ignorance of the whole subject of oral prophylaxis; it is an example of the futility of theory in dentistry *vs.* practical experience.

In reply to the seeming objection respecting "the non-receptiveness of the mass of the people," it may be said, the statement is assertion only; it has no basis in fact.

To "the mass of the people" dentistry of the present extends no benefits, it presents no hope, it affords no relief, save in the one direction,—that of ridding the mouth of aching teeth through extraction. "The mass of the people" are not instructed respecting the teeth; they are wholly unacquainted with the necessities, requirements, or value of these organs. How, then, should they be expected to follow out any theory or give attention to the details of any "system," for their betterment or preservation?

This, however, is true: whenever requisite presentation respecting the infectious states of the teeth in ordinary conditions of the human mouth is made to people of intelligence, with whom dentistry has largely to do, and the relations of these conditions to decay of the teeth has been correlated and explained, the liveliest interest has always been awakened, and patients have been stirred to willing co-operation in all reasonable efforts for the betterment and immunizing of the teeth by the system of prophylaxis treatment I have advocated for the six years just past.

When instructed, patients have been quick to see and appreciate the imminent danger of systemic infection due to septic states of untreated teeth. A set of septic teeth, ordinarily presenting a surface of twenty to thirty square inches in the oral cavity, a cavity which has been aptly designated the vestibule of life, is a perpetual menace to human health.

No adequate consideration has ever been given to the sources of infection inherent in the human mouth, consequent upon the presence of natural teeth. The adverse *local* consequences—decay, alveolar necrosis, pyorrhœa, gum recession, and final loss of the teeth—are as nothing in comparison with the evils to be revealed in the systemic disturbances and diseases which result from the continuous state of infection found in association with the teeth in the human mouth. These conditions, studied by dentistry and intelligently set forth to "the mass of people" would find "a receptiveness" which would not only astonish the opponents of this "system" of preventive treatment, but would render incalculable benefits to general health, longevity, and the happiness of all civilized humanity.

WHAT THE PROPHYLAXIS TREATMENT IS.

Let us here consider the nature and character of the prophylaxis treatment, and what it does for the mouth and teeth.

In general the treatment consists of enforced, radical and frequent change of environment for all teeth and all mouth conditions, and the maintenance of perfect sanitation for the oral cavity. More in detail it is the careful and complete removal of all concretions, calcic deposits, semisolids, bacterial placques, and inspissated secretions and excretions which gather on the surfaces of the teeth, between them, or at the gum margins; this instrumentation to be followed *in every case* by the thorough polishing of all tooth surfaces by *hand methods,—power polishers should never be used,*— not alone the more exposed labial and buccal surfaces of the teeth, but the lingual, palatal, and proximal surfaces as well, using for this purpose orange-wood points in suitable holders (porte polishers) charged with finely ground pumice-stone as a polishing material. Treated in this manner the teeth are placed in the most favorable condition to prevent and repel septic accumulations and deposits, and what is of equal importance, it aids the patient in all efforts to maintain sanitation and cleanliness.

"Brushing the tongue" for the removal of infectious coatings has been advocated more recently. Tongue *brushing* is impractical and devoid of utility. A coated tongue, especially if accompanied with tonsular inflammation, may very properly and advantageously be disinfected (mopped off) by the use of germicidal applications on bibulous paper. I have produced marked beneficial results, when finding the tongue infectiously coated, through mopping it off once or twice a day with phenol sodique. There are other remedies for this purpose perhaps equally efficient.

SOME SPECIFIC BENEFITS.

Studying more closely some of the specific benefits which result from the oral prophylaxis treatment, we notice first those that accrue to the teeth themselves. Three to six months of this treatment consistently carried forward will effectually change the whole appearance, and to some extent the whole character of a set of teeth. This change is seen in all cases, young and old, but it is specially marked in children and youth and in young adults. The dull, opaque, and lifeless aspect of the teeth exhibited in ordinary conditions of nearly all mouths, quickly gives way, under the prophylaxis treatment, to a clear, pure tooth color; the teeth in a limited time become naturally translucent, and present the appearance and true characteristics of living, healthy organs. Teeth that appear as a disfigurement in the mouth, even to disgust and loathing, when subjected for a time to the prophylaxis treatment, become strikingly ornate and attractive. The osseous structures, after a few months, exhibit unmistakably a marked change for the better, and in most cases they become wholly immune to decay. The vital forces within the tooth—the pulp—and those surrounding it—the pericementum and its connections—are stirred and stimulated to new life and activity. Circulation in dentine and enamel is revived and quickened; old, stagnant colors and deposits are manifestly taken up and removed, and new, fresh material deposited in place of them.

It is most interesting and instructive to witness the awakening and revivifying of the life forces of the teeth under treatment, as exhibited in the discharge of this undue and disagreeable color, in the brightening general aspect, and in the cessation of hypersensitiveness and irritability.

An irritating, life-destroying infection continuously adherent to the surfaces of untreated teeth, becomes a condition of violent exhibition in some cases, and when trained to recognize it, it is distinctly manifest in all. This infection retards circulation, hinders nutrition, and greatly interferes with the general health of the teeth. Manipulative treatment and the medication attending its frequent removal stimulates and fosters the remarkable changes and improvements noticed in the character and substance of all teeth which are the subject of this treatment.

The prophylaxis treatment, as yet but very imperfectly understood even by its friends, has been charged as merely a form of "tooth cleaning." Far more than this, it is a manipulative process that positively relieves the teeth from a virulent infection, and introduces a stimulation most beneficial to their internal and external life. If a tooth-cleaning process, it is one of profound significance.

If time would permit, instances might be specifically cited and multiplied in which *no* new decay has appeared in mouths, even with complete sets of teeth, for four, five, and six years; and many others in which but a modicum of decay has been found in the same length of time, and that chiefly around old fillings.

### WHAT IT DOES FOR DECIDUOUS TEETH.

The deciduous teeth in all cases are quickly and markedly influenced by the treatment. Of the limited number of children under seven years of age who are under the prophylaxis treatment, not one presents a new decay or a new defect in a tooth.

AN INTERESTING CASE.

One remarkably interesting and instructive case is that of a boy, of strongly marked nervous temperament, brought to me when but three and a half years of age, in delicate health. At this time there were five large cavities—three approximal—in his teeth, and two places exhibiting such predisposition to decay that I prognosed cavities in them within three months. Through exercise of patience and perseverance the cavities were excavated and filled with amalgam. (Perfect operations were impossible.) Immediately following the filling, for an entire year, his teeth were carefully treated every two weeks, barring one month during the summer vacation. Since that time—nearly five years—I have seen him on an average once a month; meanwhile, his teeth have received rather more than the ordinary care of childhood, at home. The boy is now in his ninth year, with this record: excessive nervousness arrested; general health fully established; teeth, as to decay, in perfect condition (predicted cavities did not appear); one new pin-head cavity in distal sulcus of left superior temporary molar (probably started before first five fillings were made); one re-decay around largest and most difficult of the five original fillings. Twelve of the temporary teeth have loosened and come out, every one in nature's own way, through complete root absorption. First permanent molars, inferior and superior permanent incisors erupted, cuspids presenting. The first molars—erupted at five and a half years—have unusually large crowns with strongly-marked, pointed cusps and correspondingly deep sulci and linear markings. Formation, shape, and time of eruption of these teeth presaged early and rapid decay; nevertheless, they are at this writing all in perfect state of preservation, and evidently improving in character. Neither infection nor chemical agents are permitted to fasten upon any of their surfaces, and the life forces are evidently building into them a more compact and decay-resisting structural consolidation.

The benefits resulting from the prophylaxis treatment as exhibited in this case are equally marked in every case where deciduous teeth have been subjected to the treatment.

Another result, convincing as to the benefits of this treatment beyond all others that can be cited, may be witnessed in connection with many cases in which decay, running riot in both temporary and permanent teeth at ten and eleven years of age, has been practically stopped following the full institution of the prophylaxis treatment. The second or twelfth-year molars, erupting into a healthy environment, have been universally found in perfect condition, and have continued thus far devoid of decay, even in the sulci. Not in one instance merely, but in practically every instance, these conditions exist.

Other and most convincing proofs may be seen in connection with the eruption of wisdom-teeth. Perhaps the most noteworthy instance in my practice in this connection is that of a young man, at the time about nineteen, for whom I had operated from boyhood, long antedating the introduction of the prophylaxis treatment. The teeth in this mouth had decayed early and in all directions. Crown, approximal, and labial fillings are to be seen in all parts of the mouth; even the lower incisors and cuspids had not escaped. The four wisdom-teeth appeared about two years after beginning an irregular and unsatisfactory course of prophylaxis treatment, but even with this, the contrast between these teeth and the twenty-eight which preceded them affords a most instructive lesson regarding the benefits of treatment. Despised, misrepresented and condemned to decay and loss, as wisdom-teeth frequently are, these teeth (and I could mention a number of other similar cases), erupting into a mouth which had been quite irregularly under treatment, were perfect. Decay had appeared in every class of teeth which developed before the prophylaxis treatment commenced, but in these wisdom-teeth there was neither decay nor the appearance of it, and this condition pertained until the case passed from under my care.

### THE INFLUENCE OF THE PROPHYLAXIS TREATMENT ON SENSITIVE DENTINE.

As a further observation of the benefits of this treatment, I desire to put on record a development of marked significance,—namely, a decided modification noticed in the sensitiveness of the dentine of teeth, especially in children, following the institution of a regular and consistent treatment. Diminishing of acute sensitiveness seems to follow as a general result, and not to be some special temporary condition. The extreme sensitiveness in the dentine of young teeth has been so modified and lessened, apparently by this treatment, that ordinary operations for filling have been performed without the intense suffering usually attending such cases. Children and young people who are under this treatment submit to ordinary operations without compulsion or special complaint.

Operations that under the old *régime* would cause much suffering, if not deemed unbearable, have been greatly mitigated in intensity.

It is an inadequate presentation to say that the prophylaxis treatment is a marked modifier of sensitive dentine, especially in the young, and that it is a mitigator of suffering under all dental operations. This may seem an extreme statement, but when fully tested by experience it will be received as conservatively true.

Hypersensitiveness of dentine is a result of pericemental irritation far more than of pulp irritation. The external and most important life of the tooth—the pericemental life—is markedly influenced by the irritative infection found always at the necks of untreated teeth. Removal of this infection is the removal of the cause of much of the undue sensitiveness of dental tissue.

### THE MARKED BENEFICIAL INFLUENCE OF THE TREATMENT IN CONNECTION WITH GUM AND PERICEMENTAL TISSUE.

The benefits which accrue directly to the teeth are not more manifest nor more pronounced than such as immediately appear in the pericementum and gums. The presence of irritative infectious matter on the surfaces of untreated teeth, sufficient to affect the pericementum and surrounding gum tissue, is found in connection with all untreated teeth. It appears coeval with their eruption, and continues, in ordinary conditions, as long as the teeth are retained. It is bounded only by the extent of the dentate surface exposed in the mouth; as a result, the pericemental tissue and gums in ordinary mouth conditions are perpetually in a state of undue sensitiveness, and not less, a condition of undue, often extreme, vascularity. Under the prophylaxis treatment, through which all irritants are removed, these tissues quickly lose their extreme and unnatural sensitiveness, and recover the normal condition of low-grade sensibility. (Gum tissue in a state of health is always without acute sensation.) The circulatory vessels also lose the unnatural distention and tumefaction consequent upon irritation, recover tonicity, and contract to normal dimensions. Three to six months of treatment will remove all marked sensibility and tendency to bleed, and operations for filling at cervical margins, especially on the upper jaw, can be safely and easily performed without the use of the rubber-dam. It is the irritative, toxic matter at the necks of the teeth that occasions the sensitive states and highly vascular conditions of the gums and pericemental tissue. A mouth with teeth freed from this infection and kept in an aseptic condition will have gums tense and hard, without vascularity and without undue sensibility.

We have, then, to commend the prophylaxis treatment: first, for its prevention of decay; second, for its stimulation to external and internal life and health of the teeth; third, for its influence in decreasing sensation in dentine and gum tissue; fourth, for its correction of the undue vascularity in gum tissue; and fifth, for its positive removal of all infection from the mouth and ultimately from the breath.

SELF-TREATMENT BY PATIENTS.

It may be well to enter somewhat into detail respecting the care and co-operation expected, *even required,* of patients under the prophylaxis treatment. In the beginning, for four to six months they are required to prèsent regularly, on appointment, once a month for treatment, which is carefully applied, using only hand methods as heretofore described. Every patient should be armed with proper appliances, especially brush and dentifrice, for cleansing the teeth and mouth, and fully instructed in the use of the same.

The tooth-brush, which is the main reliance of the patient, is at best an inefficient instrument for cleansing teeth. It is, however, the best our civilization has produced, hence until something better is devised, necessity compels the use of it: of the hundreds of shapes, varieties and kinds on the market, very few are suitable for use. Unfortunately the choice and *selection* of tooth-brushes for the general public is now largely relegated to salesladies in the department stores, and the preparation of dentifrices and so-called mouth-washes is in the hands of druggists; a condition of things as incongruous as is the compounding of medicines by the post-office clerk in a general country store. Would not dentistry itself be greatly embarrassed, if not awkwardly handicapped, if suddenly confronted with a demand, from kingly authority, for a really efficient tooth-brush or dentifrice? Probably not one in a thousand could present a clear, consistent idea of what the character or shape of a brush should be to best do the work of cleansing the teeth.

It may be said, without hesitation or fear of contradiction, there is no part of the human body which is so imperfectly and ignorantly cared for as this most important cavity containing the teeth. Attending to the teeth by the patient is a matter which has neither been studied nor taught either by the professionalist or the laity.

The child is *taught* to wash the hands, to bathe the body,—but the human mouth, the very vestibule of life, is left wholly without intelligent care.

Witness the education of the average child in a matter of such vital importance: After breakfast (occasionally), "John (or Mary), have you brushed your teeth this morning?" "No, ma'am." "Well, go right away to the bath-room and brush your teeth." Has the child ever been taught rational methods? No. Examine the equipment placed in the hands of childhood for this important operation. Even when new, a cheap, ill-adapted brush and unusable dentifrice (good enough for the children) is all they can find. This want of intelligent instruction, and these careless methods in childhood, follow through life.

The results are everywhere seen in repulsive exhibits of decayed, infection-coated teeth, offensive breaths, a display of discolored fillings, and that dental monstrosity, so common,—the gold crown.

Solomon said of the teeth of the daughter of Zion, the perfection of beauty, "Thy teeth are like a flock of sheep, even shorn, which go up from the washing whereof every one bearest twins, and there is not one barren among them." A most beautiful description of a perfect, thoroughly cared-for set of teeth.

The general public, compelled to rely on the tooth-brush, is yet without proper equipment for cleansing the teeth. The whole operation soon becomes distasteful, irksome, and generally the merest farce. Patients present with mouths reeking with infection, teeth loaded with deposits that are antiquated, and affirm "that they are very careful about brushing their teeth."

All this should be changed. It can only be done by a process of education, and that through instruction given by dentistry. Every dentist should first inform himself, and then fully instruct individual patients in the details of the care of the human mouth. The people should be taught that cleaning the teeth is not merely the *possession* of a tooth-brush. The best-shaped brush in the world will not clean the teeth in a human mouth unless it is intelligently used.

A tooth-brush should possess the requisites of shape, substance, and stability. In shape it should be plain and perfectly straight, the bristles neither coarse nor fine, set in rows of different lengths; in substance, all of the best materials. There should be no concavity to fit the arch when the brush is at rest; no fancy curves; no tufts of bristles at the end. It should be of size, in length and width, to form a mass of bristles to cover the teeth and give stability and substance when in use.

In using the brush it should be passed over labial, buccal, lingual, and palatal—or the whole outer and inner surfaces of the teeth, with a vigorous *horizontal* movement, the hand adapting the brush, as far as possible, to all tooth surfaces. With care and a straight brush all parts of the exposed crowns may thus be reached and cleansed as well as a brush can do this office work. Attempting to cleanse the teeth with the vertical movement, using a concave brush, as some teach, on the convex surface of the dental arches, while of little avail, will do no harm, either to the teeth or gums. Such use of the brush in a measure relieves the interstices of food remains, and thus assists in a work that can be much better done with a tooth-pick. The most widely advertised, and perhaps the most popular tooth-brush of the present, is the so-called "prophylactic." This brush, and all similar in shape, is a conspicuous example of lack of adaptation of means to an end. It demands the vertical movement of the brush on the external surface of the arches and on the inner surfaces it presents a double bow, most effectually preventing contact of any considerable surface of the brush with the most uncleanly portions of the teeth. A plain brush, with horizontal movement, is much better and much more efficacious.

It has been urged that the vertical movement of the brush acts as a kind of massage, tending to brush the gums onto the teeth, while the horizontal movement brushes them away from the teeth. This is the merest *theory*, a statement wholly without justification in actual conditions. The vertical movement of the brush is not more a massage of the gums than is the horizontal movement. There is no process of brushing which approximates a massage treatment of the gums, and it would avail nothing if there could be. My patients are always instructed, in connection with the prophylaxis treatment, to use the brush with a vigorous horizontal movement, even when the gums and alveolar process have necrosed until the festoons have become everted, and to continue the brushing until the teeth are not only brushed but *cleansed*. I have never yet seen a case in which reformation of tissue has not followed the treatment, and in all cases not connected with pyorrhœa, restoration of the festoons has been complete and perfect.

The tooth-brush implies a dentifrice, and a dentifrice is demanded for cleaning the teeth. To attempt a review of the compounds under the head of so-called dentifrices would consume far more time than is allotted this paper. We can only say of the tooth-powders, tablets, washes, and pastes sold as dentifrices, while they are manufactured and sold largely through the greatest empiricism, and while some are far more efficient and pleasant than others, few of them can be classed as positively harmful when used within proper limits.

So-called tooth-powders are compounded from some base, generally prepared chalk (*calc. carb. precipitas*), orris root (*iridis Florentinæ*), acidum salicylicum, or some other material. To the base selected may be added, according to the fancy of the druggist or compounder, white sugar, soap, soap-bark, cinchona, cinnamon, cuttle-fish bone (*ossis sepiæ pulveris*), pumice-stone (*lapidis pumicei pulveris*), flavoring and coloring, or whatever may be supposed to make a pleasant and salable preparation, to be advertised and sold "for beautifying the teeth and imparting sweet odors to the breath." We are not altogether decrying these powder preparations, for something of the powder or soap nature is demanded always with the brush. Patients should be taught to avoid all pastes or liquid dentifrices, for they are not only inefficient, but frequently harmful. A good soap, as a pure Castile, may be used *ad libitum*, and always with benefit to the mouth.

The impression very generally prevails that the *time* for brushing the teeth is after meals. This is a fallacy for dentistry to uproot. To receive the greatest benefit from the use of the tooth-brush, the mouth should be thoroughly cleansed always just *before* meals. Infection gathers upon the teeth in the interim between meals, when the salivary glands are at rest and the teeth not in use; it is during periods of rest that the mucous secretion and mucoid excretions from mucous surfaces are lodged upon the teeth in greatest quantity. Cleansing the mouth and teeth from this viscid débris *before* meals prevents the washing of these toxic tooth accretions into the gastro-intestinal tract with the food. Who is not taught to wash the *hands* before meals? Of how much greater importance is the cleansing of the human mouth! If the accustomed method with the brush is not practicable, it may be always practicable to thoroughly rinse the mouth with water and to wipe the main exposed surfaces of the teeth with the corner of a towel or napkin. This method of cleaning the teeth is not enough in vogue. After meals the teeth may be freed of food remains with some form of tooth-pick, preferably the "quill," which is all that is required. Careful cleansing, devoting not seconds but minutes to the operation, should always precede retiring for the night and immediately succeed rising in the morning.

Cleansing the mouth thus frequently, and at stated periods, will greatly tend to preserve the teeth from decay, and surely lessen the inroads of systemic infection.

ANOTHER VIEW OF MOUTH INFECTION.

The photographer who would make sure of a satisfactory picture, having made one negative, will frequently change the position of his subject for another view; he may move the head into another light, readjust the camera and screens, or perhaps rearrange the shading. That we may fasten upon the mind and carry away with us a more vivid and lasting picture of these adverse, repugnant and hurtful mouth conditions, let us here endeavor to secure a picture from another point of view.

A Mr. McC. quite recently applied for relief from one of the most unpromising conditions of pyorrhœa it has been my good fortune to encounter. I say "good fortune," for I regard it as good fortune that through study and practice of the oral prophylaxis treatment the dental profession is now able to throw off the shackles which have so long held it in bondage to the commonly accepted theory that alveolar pyorrhœa is a *disease* of constitutional origin, and incurable. It is good fortune as well as a great pleasure to bring relief to a long list of afflicted ones, and to proclaim the fact that alveolar pyorrhœa is not a *disease* in the true meaning of that term; that it does not result from the gouty or uric acid diathesis; it is a pleasure to proclaim that it is not a result of some "constitutional vice," nor of any special constitutional condition. The glamour and charm of mystery no longer hang over this affection; the light of rational, indisputable, scientific truth shines through the mists and clouds of speculation and theory which have so long enveloped it.

WHAT ALVEOLAR PYORRHŒA IS.

Alveolar pyorrhœa is not properly classified as a "disease." Its very definition—flow of pus—implies that it should be taken from the category of diseases and placed among the inflammations. Properly defined, alveolar pyorrhœa is an inflammation within the confines of the pericemental membrane and contiguous tissues; an inflammation due wholly to the irritation resulting from stagnant septic matter adherent to the surfaces, necks, and roots of natural teeth. It need no longer be said that these are "constitutional conditions, complex in their manifestations;" neither that their "medical and hygienic management are almost exclusively in the hands of the physician." How utterly absurd is the proposition that the "duty of the dental practitioner is confined largely to the question of diagnosis." Treatment of pyorrhœa relegated to the medical profession! *Neither* diagnosis nor treatment belongs to medicine; it is a mouth disorder strictly within the realm of the dental practitioner. It is to the discredit of dentistry and not of medicine that such inconsiderate and contradictory teachings prevail respecting it. It is a mouth affection in association with the teeth of which medicine, as a profession, has never assumed to know. Occurring in mouths with large, strong, and apparently healthy teeth, how should medicine suspect that it is largely, if not entirely, the prime cause of renal disturbances, uræmia, diabetes, and albuminuria; and many other constitutional disturbances of which we know so little?

I here exhibit casts of the mouth referred to as it presented from the hands of one of the fashionable dentists of Philadelphia. It is a typical case of pyorrhœa. A married man in middle life—thirty-eight years—with hard, strong teeth, crowns large and irregular, roots not long and probably conical, marked cervical constriction, and pericemental tissue scanty. This gentleman is a smoker, but not given to excesses or vices; had brushed his teeth latterly with so-called "prophylactic" brush after approved methods, vertical movement.

As through this case we look upon the horrible conditions in human mouths everywhere, in high and low born, king and peasant, and realize that they are wholly due to states and conditions of natural teeth, and so largely preventable, shall not we rise as witnesses for, and to advocate better things for, neglected, untaught, suffering humanity?

But to return to this typical case of pyorrhœa: The broad-bladed scaler of the Smith set was passed back to the lingual surface of the second lower molar, where it was loaded with years of accumulations of calcic toxins, recent exudations of globules of pus, and gatherings of every variety of mouth débris. This conglomerate mass of revolting matter was held up to view, and the patient asked if he would like to handle it, to smell it, to taste it, to have it returned to the mouth. To all of these suggestions there was prompt declination. Then it was clearly explained that matter of similar nature is found on all untreated teeth, and that teeth are not on the outside of the body, *but inside the mouth;* that such conditions exist, not one hour in the day, but twenty-four hours of every day; not one day in the year, but three hundred and sixty-five days of every year; and further, this infectious matter on the teeth, *in the mouth,* is in a temperature constantly maintained at 98.6° F., than which nothing could possibly be more favorable for germ culture, for imparting odors to the breath, or for filling the lungs with infectious emanations.

From the mouth and teeth, septic matter is being conveyed directly into the gastro-intestinal tract, thence to the circulation, whence it may be deposited in any organ or tissue, and become the nucleus of serious chronic, systemic maladies. To emphasize the continuous presence of this deleterious matter, the patient was told he could at any hour of the day draw floss silk between the teeth, or pass the finger over their surfaces, and discover most repellent odors; or he could recall the unpleasant taste in the mouth on rising in the morning, the fetid breath, especially at night,—due not to stomach conditions, but to the stagnant, odoriferous matter on the teeth. Becoming dry, through inactivity of the salivary glands, the teeth send out into the breath of the sleeper poisonous emanations to do continuously a work of infection, unnoticed and unsuspected.

I would that it might be recognized by all, that sequences of the universal infection on the teeth in the oral cavity are, in our present civilization, affecting the health of humanity to a greater extent than any other one physical condition. I could wish nothing grander or better for our profession than that it should see and embrace the magnificent opportunity unfolded in oral prophylaxis to move forward and occupy this new field of untold benefits.

Dentistry, in these revelations respecting states of infection ordinarily existing in the human mouth, has opened a door for general etiological research of far-reaching importance.

The secrets of tooth decay, in and of themselves, are of minor consequence in comparison with the systemic infections due directly to states and conditions of the teeth in the mouth. We venture to predict that recognition will ere long be made of the fact that tuberculosis,—that decimator of homes,—and possibly other grave chronic disorders, can find no soil for development in a system kept from the contaminations of mouth-infection through a rigorous prophylaxis treatment from childhood.

We have abundantly proved that diabetes and many gastro-intestinal troubles are directly traceable to the mouth-infection of alveolar pyorrhœa; also that many pharyngeal and tonsular inflammations, and many skin troubles, have their origin in infection in the mouth, due to septic states of neglected teeth. We know, also, that mental depression and hypochondria, and many of the perplexing nervous conditions in women, result from the same cause.

Dentistry alone has the power to relieve humanity from the plague of mouth-infection. When shall it command its own rightful place among the specialties of medicine?

